# The Role of Preventive Nutrition in Chronic Non-Communicable Diseases

**DOI:** 10.3390/nu11051074

**Published:** 2019-05-15

**Authors:** Nicola Di Daniele

**Affiliations:** UOC of Internal Medicine, Center of Hypertension and Nephrology Unit, Department of Systems Medicine, University of Rome Tor Vergata, via Montpellier, 100185 Rome, Italy; didaniele@med.uniroma2.it; Tel.: +39-06-2090-2982; Fax: +39-06-2090-3362

**Keywords:** preventive nutrition, chronic non-communicable diseases, obesity, metabolic syndrome, Mediterranean Diet, arterial hypertension, chronic kidney disease

Over the last century, there has been a substantial change in the lifestyle and dietary habits of people worldwide. This change, however, is not reflected to the same degree in our genetic makeup, resulting in an imbalance between calorie intake and energy expenditure, hence leading to overweight and obesity both in adults as well as in adolescents. According to a recent World Health Organization (WHO) report, obesity is almost tripled since 1975 [1]. In 2016, 41 million children under five years were found to be overweight or obese, creating an important global public health issue as “an obese child is likely to become an obese adult” [2].

Furthermore, we must also consider that overweight and obesity are relevant risk factors for chronic degenerative non-communicable diseases (NCDs) (such as arterial hypertension, type 2 diabetes mellitus, cardiovascular diseases, metabolic syndrome and cancer) [3].

In this regard, *Behavioural Therapy* can be very useful in the prevention and treatment of obesity when combined with a substantial change in dietary habits and an increase in physical activity [4]. This kind of intervention becomes significantly more effective when treating children since it has been amply demonstrated that it is more difficult to treat adult obesity than childhood obesity through changes in lifestyle and eating habits alone [5]. Despite that the underlying molecular mechanisms require still clarification, several descriptive studies have shown that a modest weight decrease of about 10% can increase life expectancy and prevent the onset of NCDs in obese patients [6,7,8]. This raises the important points of both an urgent basic research in the field, as well as an immediate pragmatic measure of intervention.

In light of what has been said so far, preventive nutrition (PN), a crucial branch of nutrition science, is particularly relevant in that it has a key role in the treatment, regression and prevention of NCDs associated with overweight and obesity. The scope of PN is in constant evolution. In fact, it not only focuses on nutritional aspects per se but also takes into consideration the qualitative and quantitative presence of the natural bioactive compounds and their potential beneficial health effects as part of a proper diet [9,10]. Since the 1950’s several studies have focused on the Mediterranean Diet (MD) as a reference food model for the prevention of NCDs, to the point of being recognised by UNESCO as an intangible Cultural Heritage of Humanity in 2010 [3,11]. 

The first study that focused on the possible benefits of PN was the “Seven Countries Study of Cardiovascular Diseases” (SCSCD) which is credited for having characterised the “Mediterranean Diet” for the first time and for having documented its protective role against coronary heart disease [12]. This nutritional model, which has its origins in various cultures and countries of the Mediterranean basin, is characterised by a high intake of fruit, seeds, vegetables, bread, cereals, extra virgin olive oil (a source of unsaturated fats) and fish, a moderate intake of white meat (poultry), eggs, legumes, dairy products and red wine and a low intake of red meat and animal fats (saturated fats) [12].

Several studies have shown that adherence to MD, that is rich in fresh vegetables, is associated with a lower incidence of NCDs [13,14]. In fact, its positive effects are evident both in primary and secondary prevention. However, the exact molecular mechanisms by which adherence to the MD exerts its protective action have still not been fully identified.

One of our studies has shown that the Italian Mediterranean Organic Diet (IMOD) has healthy effects on pre-obese and obese patients with normal renal function and on chronic kidney disease patients (CKD) stage II-III (according to K-DOQI guidelines) [15], inducing an improvement in body composition, reducing fat mass both in Kg and % and increasing lean mass in % [16]. Fat mass reduction is directly related to the risk of developing type 2 diabetes mellitus and cardiovascular diseases secondary to low-grade chronic inflammation state which is typical of obesity and metabolic syndrome. Moreover, a reduction in body weight has a significant impact on the risk of developing cardiovascular diseases with complications that can lead to premature death. Accordingly, it has been observed a significant reduction of plasma homocysteine levels, a risk factor for cardiovascular diseases that is directly involved in atherosclerotic processes [16,17,18].

Many randomized controlled clinical trials have demonstrated that eating habits, together with physical activity, can modify the predisposing factors of NCDs, such as low-grade of chronic inflammation, the epigenetic and transcriptional mechanisms and the alterations of the circadian rhythms [19]. For this reason, the combination of regular physical activity and a balanced diet has a central preventive role in reducing the incidence of NCDs. 

In a recent study, a method has been developed to define a series of age-related illnesses; more specifically those with an incidence rate among adult population, which grows with the square of age, as well as to evaluate the impact that these illnesses could have on the subject’s health status. This method allows us to not only evaluate the chronological age, but the psycho-physical well-being of the elderly person, the existence of any form of disease and their severity. Consequently, by using this method, it will be easier to identify the main modifiable health risk factors of aging, among which PN and physical activity deserve special consideration [20].

In conclusion, it is clear that PN has an important role on a global scale. In fact, if PN is introduced at the right time and with the right methods, it is a fundamental tool for the prevention of NCDs, leading to a significant reduction in the mortality rate of patients affected by these conditions and having a positive impact on the reduction of public health spending [21,22,23]. 

Not last, I would like to thank all the authors and reviewers who contributed to the success of this special issue that I edited. A special thanks goes to the Nutrients team for the prompt and efficient help they gave in the preparation of this special issue.
